# Endothelial Progenitor Cell Dysfunction in Cardiovascular Diseases: Role of Reactive Oxygen Species and Inflammation

**DOI:** 10.1155/2013/845037

**Published:** 2012-12-30

**Authors:** Chih-Pei Lin, Feng-Yen Lin, Po-Hsun Huang, Yuh-Lien Chen, Wen-Chi Chen, Huey-Yi Chen, Yu-Chuen Huang, Wen-Ling Liao, Huey-Chun Huang, Po-Len Liu, Yung-Hsiang Chen

**Affiliations:** ^1^Department of Biotechnology and Laboratory Science in Medicine and Institute of Biotechnology in Medicine, National Yang-Ming University, Taipei 112, Taiwan; ^2^Department of Pathology and Laboratory Medicine, Department of Internal Medicine and Divisions of Biochemistry and Cardiology, Taipei Veterans General Hospital, Taipei 112, Taiwan; ^3^School of Medicine and Cardiovascular Research Center, National Yang-Ming University, Taipei 112, Taiwan; ^4^Department of Internal Medicine, School of Medicine, Taipei Medical University and Cardiovascular Research Center, Taipei Medical University Hospital, Taipei 110, Taiwan; ^5^Faculty of Medicine and Institute of Clinical Medicine, National Yang-Ming University, Taipei 112, Taiwan; ^6^Department of Anatomy and Cell Biology, College of Medicine, National Taiwan University, Taipei 100, Taiwan; ^7^Graduate Institute of Integrated Medicine, School of Chinese Medicine, College of Chinese Medicine and Department of Medical Laboratory Science and Biotechnology, College of Health Care, China Medical University, Taichung 404, Taiwan; ^8^Departments of Urology, Obstetrics and Gynecology and Medical Research, Genetics Centre and Center for Personalized Medicine, China Medical University Hospital, Taichung 404, Taiwan; ^9^Department of Respiratory Therapy, College of Medicine, Kaohsiung Medical University, Kaohsiung 807, Taiwan

## Abstract

Endothelial progenitor cells (EPCs) move towards injured endothelium or inflamed tissues and incorporate into foci of neovascularisation, thereby improving blood flow and tissue repair. Patients with cardiovascular diseases have been shown to exhibit reduced EPC number and function. It has become increasingly apparent that these changes may be effected in response to enhanced oxidative stress, possibly as a result of systemic and localised inflammatory responses. The interplay between inflammation and oxidative stress affects the initiation, progression, and complications of cardiovascular diseases. Recent studies suggest that inflammation and oxidative stress modulate EPC bioactivity. Clinical medications with anti-inflammatory and antioxidant properties, such as statins, thiazolidinediones, angiotensin II receptor 1 blockers, and angiotensin-converting enzyme inhibitors, are currently administered to patients with cardiovascular diseases. These medications appear to exert beneficial effects on EPC biology. This review focuses on EPC biology and explores the links between oxidative stress, inflammation, and development of cardiovascular diseases.

## 1. Introduction

The vascular endothelium is a key feature in vascular homeostasis. Endothelial dysfunction and injury are considered to be the first steps in atherogenesis [1–4]. Recent studies indicate that endothelial dysfunction and injury in the vascular wall are repaired by bone-marrow- (BM-) derived endothelial progenitor cells (EPCs) [5]. Evidence suggests that CD133^+^CD34^+^KDR (vascular endothelial growth factor receptor 2, VEGFR2)^+^-EPCs are mobilised from the BM into the peripheral blood in response to tissue ischemia or injury [6]; these cells migrate to sites of damaged endothelium and differentiate into endothelial cells (ECs) [7], thereby improving blood flow and tissue repair [8, 9]. EPCs contribute to reendothelialisation and neovascularisation. Their beneficial effects may be mediated through paracrine secretion of angiogenic factors and cytokines. Several lines of evidence indicate that EPCs constitute an important endogenous system that maintains endothelial integrity and vascular homeostasis [8]. Patients with cardiovascular diseases, such as coronary artery disease (CAD), hypertension, heart failure, and diabetes [10], exhibit reduced EPC number and function [11]. Therefore, reduced EPC levels may reflect a mechanistic link that confers increased risk of adverse cardiovascular outcome. Reversal of EPC dysfunction could therefore potentially prevent the progression of cardiovascular and vascular disease [12].

In various forms of cardiovascular disease, inflammation mediates oxidative stress [13], dysfunction, injury, and senescence (cellular aging) of ECs [14, 15]. Inefficient recruitment of EPCs results in vascular dysfunction and accelerates the progression of cardiovascular diseases [16]. Augmentation of vascular repair by the provision of growth factors such as vascular endothelial growth factor (VEGF) or by direct migration of EPCs into the endothelium can be protective and prevent ongoing vascular damage. Because reendothelialisation or neovascularisation is a pro-inflammatory response, this process becomes self-sustaining [9]. Recently, it has become increasingly apparent that these changes occur in response to oxidative stress [17], possibly as a result of systemic and localised inflammatory responses [18]. The interplay between inflammation and oxidative stress is involved in the initiation, progression, and complications of cardiovascular diseases [19]. Evidence from recent studies suggests that inflammation and oxidative stress modulate EPC bioactivity [20].

A clear understanding of EPC biology is of particular relevance to cardiovascular diseases, as it may provide additional insight into the pathogenesis of these diseases, as well as novel targets for therapeutic agents [15, 21]. Recent studies propose the existence of a dynamic association between inflammation [22], oxygen-free radicals (reactive oxygen species (ROS)), and EPC biology, implying that EPCs may play a key role in vascular repair under pro-atherogenic conditions [23]. Clinical medications with anti-inflammatory and antioxidant properties, such as statins, thiazolidinediones, angiotensin II receptor 1 blockers (ARBs), and angiotensin-converting enzyme inhibitors (ACEIs), are currently administered to patients with cardiovascular diseases. These medications exert beneficial effects on EPC biology [21]. This paper focuses on EPC biology and explores the links between oxidative stress, inflammation, and development of cardiovascular diseases. A better understanding of the inflammatory and oxidative mechanisms leading to decreases in the numbers of EPCs and functional impairment of EPCs may provide additional insight into the pathogenesis of cardiovascular disease and lead to the development of novel therapeutic strategies.

## 2. EPC Biology

**Figure 1:**
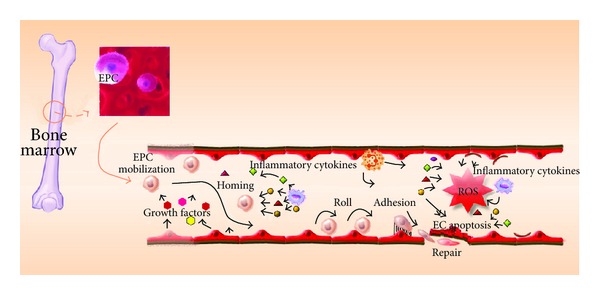
Illustration of the ability of circulating EPCs to mediate vascular endothelial cell repair.

The local BM microenvironment, the stem cell niche [24], plays a pivotal role in the mobilisation of BM-derived stem/progenitor cells [25]. Growth factors and cytokines induce mobilisation of stem/progenitor cells with various proteinases [6]. After mobilisation, homing is the first process stem/progenitor cells undergo. This process is fairly rapid [26]. Adhesion molecules mediate rolling and adhesion of homing cells to the blood vessel wall [27]. EPCs find their way to the injured endothelium via a complex signalling network for reendothelialisation (Figure 1) [28].

The other EPC function is neovascularisation. Angiogenesis and vasculogenesis are the major forms of postnatal neovascularisation. Angiogenesis is the process of formation of new vessels from preexisting blood vessels. Vasculogenesis is the process of blood vessel formation via *de novo* production of EPC-derived ECs, which in turn form blood capillaries [29]. Neovascularisation is an important process in functional recovery from pathological conditions, such as wound healing and ischemic diseases. Hypoxia is an important driving force for neovascularisation under various ischemic conditions [30]. Hypoxia stimulates expression of many cytokines and growth factors such as VEGF, platelet-derived growth factor, insulin-like growth factor, and fibroblast growth factor (FGF), which play critical roles in induction of neovascularisation [31]. Other cellular components, including monocytes, T cells, neutrophils, and platelets, also play significant roles in induction and modulation of neovascularisation. Preclinical studies have showed that EPCs with or without combination of growth factors induce neovascularisation in ischemic tissues [32, 33].

**Figure 2:**
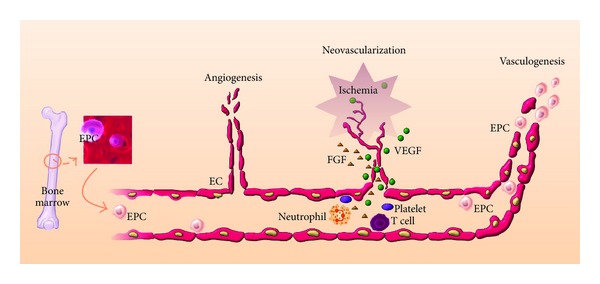
Illustration of the ability of circulating EPCs to mediate neovascularisation in ischemic tissue.

In the context of EPC biology, vasculogenesis enables *de novo* formation of vessels via *in situ* migration, proliferation, differentiation, and/or incorporation of BM-derived EPCs into the regenerating vasculature [34]. BM-derived EPCs can localise to vascular structures during skeletal and cardiac ischemia [34, 35], wound healing [36], tumor growth [37], and corneal neovascularization [38]. EPCs also produce various proangiogenic cytokines and growth factors, promoting proliferation and migration of preexisting ECs, activating angiogenesis, and contributing to vascular regeneration and reestablishment of tissue homeostasis [39]. Thus, EPCs may work not only through the activation and support of vasculogenesis but also through the activation and mediation of angiogenesis (the process of new vessel formation)[40, 41]. The paracrine aspect of EPC activity, which reflects an indirect contribution to neovascularisation, was confirmed by several reports that demonstrated the presence of various cytokines and other secreted proangiogenic factors in EPCs (Figure 2) [42, 43].

The close relationship between inflammation and oxidative stress is now well defined [44, 45]. Therefore, it is important to distinguish whether the observed effect of oxidative stress on EPC mobilisation and functional status is independent of inflammatory status. Several clinical conditions, characterised by both increased inflammation and oxidative stress, are associated with reduced numbers and impaired function of EPCs [21]. Moreover, the availability and function of EPCs are also adversely affected by risk factors for cardiovascular diseases, including hypertension, diabetes, cigarette smoking, physical inactivity, homocysteine levels (HCY), and aging [3]. For example, the hypertension pathophysiology involves a complex interaction of multiple vascular effectors including the sympathetic nervous and renin-angiotensin-aldosterone systems and the inflammatory mediator activations. Oxidative stress and endothelial dysfunction are consistently observed in hypertensive subjects, but emerging evidence suggests that they also have a causal role in the molecular processes leading to hypertension. Current understanding of the molecular mechanisms in the development of hypertension with an emphasis on oxidative stress and endothelial dysfunction is important [46]. Thus, indirect evidence suggests that inflammatory status and oxidative stress, which are related to the pathophysiology of the above-mentioned conditions and diseases, can adversely influence the number and functional capacity of EPCs [18].

## 3. EPC Dysfunction and Inflammation

Inflammation is implicated in the pathophysiology of various cardiovascular diseases [47]. Proinflammatory cytokines stimulate the expression of adhesion molecules on endothelial surfaces, promoting initiation of atherogenesis [4]. In parallel, these inflammatory factors stimulate the production of growth factors such as VEGF and mediate tissue repair [47]. EPCs are released into the peripheral circulation after stimulation by an inflammatory response and participate in tissue repair [23]. Animal studies show that EPCs are rapidly mobilised after vascular injury, in response to increased circulating VEGF levels, and contribute to neovascularisation of injured tissues [48]. Several inflammatory factors [49], such as tumour necrosis factor-*α* (TNF-*α*) [50], interleukin-1*β* [51], granulocyte macrophage-colony stimulating factor, and stromal-derived factor-1, modulate EPC mobilisation, recruitment, and homing [52–54]. Increasing evidence indicates that a transient restricted inflammatory response may stimulate EPC mobilisation, while persistent or excessive inflammatory stimuli may have deleterious effects, resulting in decreased numbers of EPCs in the circulation [5]. In addition, inflammatory factors and numbers of EPCs may increase in response to deficient endothelial regeneration, leading to vascular repair. Initial observations from animal studies suggest that inflammation induces mobilisation of EPCs. A positive association between C-reactive protein (CRP; an acute-phase inflammatory protein produced by the liver) levels and circulating EPCs has been documented in patients with stable CAD, suggesting that a systemic inflammatory state stimulates EPC mobilisation in these patients [55]. Although these observations indicate that EPC mobilisation is closely correlated with variations in the levels of some inflammatory factors in humans, there is no clinical evidence suggesting a causal relationship between inflammation and EPC mobilisation [21].

On the other hand, considerable evidence suggests that high-grade, extensive inflammatory stimulation may have the opposite effect on circulating levels of EPCs. Evidence also suggests that systemic inflammation induces EPC dysfunction in humans. Clinical studies have demonstrated that CRP is associated with senescence of EPCs in preeclampsia patients [56]. In addition, CRP exerts direct inhibitory effects on EPC differentiation and survival [57], whereas EPCs exposed to CRP exhibit decreased angiogenic activity [58]. High levels of TNF-*α* and glucose contribute to a reduction in EPC number [59, 60]. EPCs that are mobilised in response to inflammatory stimulation may be functionally impaired [61]. Indeed, ample evidence suggests that functional activity of EPCs is significantly impaired in the presence of high inflammatory stimulation, as in cases of heart failure. This leads to reduced migratory capacity and impaired clonogenic potential in EPCs [62].

The basic and clinical evidence supports the hypothesis that inflammation leads to functional impairment of EPCs, but inflammation seems to play a dual role in EPC mobilisation. Low-grade inflammation induces EPC mobilisation, whereas high-grade and prolonged inflammatory stimulation has the opposite effect [21]. Although the mechanisms regulating this effect are still unclear, prolonged exposure of BM to increased proinflammatory stimulation may lead to exhaustion of the EPC pool. This would eventually lead to release of fewer functional EPCs and the release of immature or dysfunctional EPCs. The existing clinical evidence supporting the association between inflammation and EPCs is largely circumstantial and observational [21]. Further clinical studies are required to elucidate the exact mechanisms by which inflammation affects EPC mobilisation and functional activity in humans.

## 4. EPC Dysfunction and Oxidative Stress 

The effects of oxidative stress on EPCs in cardiovascular disease are now well documented. Oxidative stress occurs when generation of ROS (or oxygen free radicals) is increased, and ROS cause oxidative damage to biological structures; this suggests that ROS play a key role in atherogenesis [63]. Oxidative stress may also play a crucial role in EPC mobilisation and functional bioactivity [64]. ROS exert a direct cytotoxic effect on the vascular endothelium [65]. Increased superoxide generation reduces EPC levels and impairs EPC function [66].

In addition to the indirect effects of ROS on EPCs, considerable evidence suggests that ROS exert direct effects on EPCs. Incubation of EPCs with high levels of hydrogen peroxide (H_2_O_2_) induces apoptosis [67, 68], profoundly reducing the numbers of EPCs [69]. In a rat model of myocardial infarction (MI), increased production of ROS is associated with reduced EPC levels [70]. Conditions associated with increased oxidative stress lead to the mobilisation of functionally defective EPCs, which have a lesser capability of mobilising, migrating, and incorporating into existing vasculature [71, 72]. However, EPCs produce superoxide dismutase (SOD), which enhances their ability to offer vascular protection [73]. Therefore, it is clear that conditions associated with increased oxidative stress not only decrease the absolute numbers of circulating EPCs but also impair EPC function, with deleterious effects on vascular homeostasis [21]. At a clinical level, increased oxidative stress status has been associated with decreased EPC mobilisation. Indeed, studies have shown that the number of circulating EPCs is associated with systemic markers of oxidative stress. Conditions associated with increased systemic oxidative stress have been associated with decreased EPC numbers in the peripheral circulation [18]. In this context, high HCY, which has also been associated with enhanced production of ROS, decreases the numbers of EPCs and impairs their function, partly through induction of EPC senescence [74].

Cellular oxidative stress, mediated by oxidised low-density lipoprotein (oxLDL), plays a key role in the pathogenesis of atherosclerosis [75]. oxLDL accelerates the onset of EPC senescence [76]. By contrast, high-density lipoprotein (HDL), which is considered to be atheroprotective partly because of its antioxidant and anti-inflammatory properties, has a positive impact on EPC physiology [77, 78]. In vascular biology, protection against oxidative stress is accomplished by a network of endogenous antioxidant defences, which exert cellular protective effects by directly scavenging ROS and reducing their damaging action. Compared with differentiated, mature ECs, EPCs from healthy volunteers express higher levels of the antioxidant enzymes catalase, glutathione peroxidase, and manganese SOD. The level of antioxidant enzyme expression in EPCs may make them relatively resistant to oxidative stress [79, 80]. However, there is insufficient clinical data to document the effects of classic risk factors on endogenous antioxidant defence systems in EPCs [81].

These studies directly implicate oxidative stress for the functional impairment and reduction in number of circulating EPCs in vascular dysfunction. Oxidative inactivation seems to play a key role in this regard. Although EPCs from healthy donors are relatively resistant to oxidative stress [4], the presence of cardiovascular risk factors may alter the redox state of these cells. The oxidative mechanisms leading to EPC dysfunction remain unclear. It is still unclear whether a direct association exists between ROS and functional bioactivity of EPCs. It is also unknown whether therapeutic strategies targeting intracellular redox states have the ability to modify the functional status and mobilisation abilities of EPCs. A definite conclusion regarding the association between ROS and EPCs can only be drawn after antioxidant therapies are shown to improve these parameters of EPC biology [82–85].

## 5. Therapeutic Strategies

 A crucial goal in the treatment and prevention of cardiovascular diseases is promotion of reendothelialisation [86, 87]. Since EPCs play a critical role in maintaining an intact and functional endothelium [8], decreased numbers of EPCs and dysfunctional EPCs may contribute to endothelial dysfunction and susceptibility to cardiovascular diseases. Improvement in the number and function of EPCs could be beneficial for patients with impaired vascular function. *Ex vivo* expansion of EPCs for therapeutic use is a promising strategy [88]. Therefore, a better understanding of the molecular mechanisms underlying the reduced number and impaired functional activity of EPCs is of potentially major significance, as it could create novel therapeutic targets in vascular disease.

As mentioned previously, EPC function is altered in the presence of excessive oxidative stress and inflammatory stimuli. Several studies have reported the beneficial effects of several medications with anti-inflammatory or antioxidant properties on EPCs, most of which are currently administered to patients for the management of cardiovascular pathologies [21]. HMG-CoA reductase inhibitors (statins) are effective lipid-lowering agents and are able to significantly reduce cardiovascular mortality and morbidity in patients at risk for cardiovascular disease [89]. Recent clinical and experimental data suggest that the benefits of statins may extend beyond their effects on serum cholesterol levels [90]. Statins increase EPC number and functional activity [91], thereby contributing to reendothelialisation of injured vessels [92]. Statins enhance nitric oxide (NO) bioavailability and exert potent anti-inflammatory and antioxidant effects beyond cholesterol reduction [93]; these properties may contribute to the favourable impact of statins on EPCs [90]. Statins stimulate EPC differentiation and survival [91] and significantly reduce H_2_O_2_-induced apoptosis of EPCs [69]. Statins have emerged as novel and powerful tools to study cardiovascular biology. The seemingly off-target properties of statins may have important clinical implications in addition to lowering serum cholesterol [94].

Angiotensin II regulates blood pressure and contributes to endothelial dysfunction and atherosclerosis progression [95]. Impaired EPC function in patients with hypertension inhibits the endogenous repair of vascular lesions and leads to the atherosclerosis progression. The number of EPCs in peripheral blood is inversely correlated with mortality and the occurrence of cardiovascular events. Angiotensin II-mediated signalling is implicated in oxidative stress, inflammation, and insulin resistance, factors that cause EPC dysfunction. Angiotensin II-mediated signalling is implicated in oxidative stress and inflammation; it accelerates the onset of EPC senescence via increased oxidative stress [16]. Blockade of the angiotensin II type 1 receptor may therefore present a new therapeutic target for enhancing EPC bioactivity [95].

Recent studies on animal models have demonstrated that ACEIs mobilise EPCs through an anti-inflammatory effect [96]. ACEIs also produce a significant increase in EPC levels in a rat model of MI [70]. At a clinical level, ACEIs enhance the functional activity of EPCs in patients with CAD, providing an additional mechanism by which ACEIs exert clinical benefits in CAD patients [97]. Similarly, treatment with ARBs also increased the number of regenerative EPCs in diabetic patients [98]. Therefore, both ACEIs and ARBs may have beneficial effects on EPCs mobilisation and functional activity, thereby contributing to the repair of injured vascular endothelium [95, 99].

Peroxisome proliferator-activated receptor (PPAR) agonists reduce vascular inflammation and oxidative stress and improve NO bioavailability [100]. PPAR-*γ* agonists promote the differentiation of angiogenic progenitor cells toward the endothelial lineage [101] and increase EPC number and migratory activity [102]. Of note, the negative effects of CRP on EPC survival, differentiation, and angiogenic function are abrogated by pretreatment of EPCs with a PPAR-*γ* agonist; this provides direct evidence of the salutary impact of anti-inflammatory agents on EPC biology [57]. More recently, PPAR-*α* was found to be essential for microparticle-induced differentiation of BM-derived EPCs and angiogenesis [103]. The precise mechanisms involved in EPC stimulation by PPAR agonists have yet to be investigated, and no data are available on humans.

**Figure 3:**
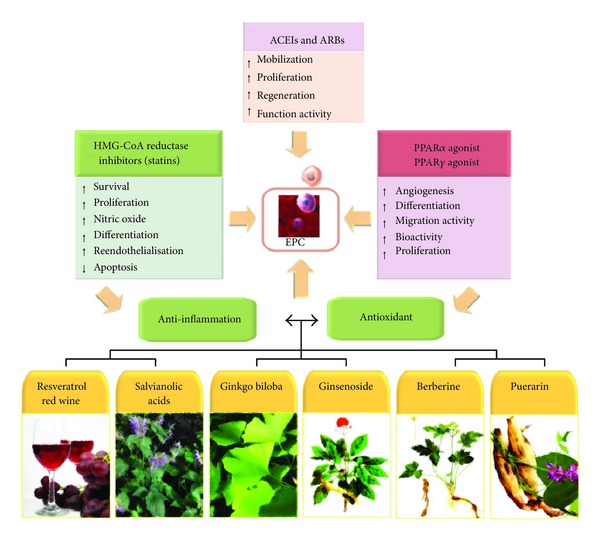
Therapeutic interventions with anti-inflammatory and antioxidative drugs/agents for functional improvement of EPCs.

In addition to clinical drugs, several antioxidative agents with anti-inflammatory properties, such as puerarin [104, 105], resveratrol/red wine [106–113], *Ginkgo biloba* [114], berberine [115], salvianolic acids [116], and ginsenoside [117], also have been found to enhance EPC bioactivity (Figure 3). In theory, decrease in oxidative stress would induce mobilisation of EPCs and improve their functionality [64]. However, there are no clinical data to support this theory in humans, and large clinical trials testing the effect of antioxidants on cardiovascular risk resulted in dramatic failures [65, 118, 119]. This therapeutic approach is hampered by the lack of effective antioxidant strategies for modification of intracellular redox states in the vascular endothelium. Therefore, when EPC mobilisation and functional status are shown to be improved after antioxidant treatment, a causal association between ROS and EPC biology will be considered significant in humans. Antioxidant strategies targeting EPC mobilisation and function may seem tempting, but it is premature to state that long-term antioxidant treatment would have any real biological value [82].

Therapeutic strategies with specific modulators of inflammatory pathways may represent more promising and effective approaches in the future. Therefore, the ability to enhance EPC number and functional capacity is a common property of otherwise diverse interventions. As these interventions act through different mechanisms and affect EPCs at different regulatory levels, they may act synergistically in optimising EPC function.

## 6. Conclusions

EPC bioactivity is affected by inflammatory and redox statuses. However, little is known about the effects of anti-inflammatory and antioxidant strategies under clinical conditions. This is a limitation of the existing literature. Most clinical studies demonstrate only associations between EPCs and vascular events. Therefore, more clinical studies are required for the establishment of EPC measurements as prognostic markers in cardiovascular disease. Nevertheless, cardiovascular diseases with increased inflammatory and oxidative stress are associated with EPC dysfunction, which is reversed upon treatment with anti-inflammatory or antioxidant drugs. Therefore, inflammatory and redox signalling pathways that interfere with EPC bioactivity should be further investigated. Additionally, clinical studies are required to determine whether these pathways are active *in vivo*. Future basic and clinical research may provide the basis for the development of novel targeted interventions to improve endothelial function and prevent cardiovascular diseases.
